# Dichotomizing partial compliance and increased participant burden in factorial designs: the performance of four noncompliance methods

**DOI:** 10.1186/s13063-015-1044-z

**Published:** 2015-11-17

**Authors:** Peter D. Merrill, Leslie A. McClure

**Affiliations:** University of Alabama at Birmingham, Birmingham, AL 35233 USA; Duke Clinical Research Institute, Durham, NC 27705 USA; Drexel University, Philadelphia, PA 19104 USA

## Abstract

**Background:**

Noncompliance to treatment assignment is an inevitable occurrence in randomized clinical trials (RCTs). Intention to treat (ITT) is generally considered the best method for addressing noncompliance in RCTs. Alternatives to ITT exist, including per protocol (PP), as treated (AT), and instrumental variables (IV). These three methods define participant compliance dichotomously, but partial compliance is a common occurrence in RCTs. By defining a threshold, above which a participant is called a complier, PP, AT and IV can be used, but the resulting loss of information may affect their performance. Trials with factorial designs may experience higher rates of noncompliance due to the heavier burden that participants experience by being assigned to multiple experimental treatments.

**Methods:**

Using simulations, we assessed the performance of ITT, PP, AT, and IV in both the partial compliance setting and in a 2-by-2 factorial design with increased participant burden for those randomized to both active treatments.

**Results:**

The bias, mean squared error, and type I error rates of the IV method after dichotomizing partial compliance were heavily inflated. The performance of all four methods depended on the level of noncompliance present, with higher average noncompliance leading to poorer performance. PP and AT showed improved bias and power relative to ITT without inflating the type I error beyond acceptable limits. However, the PP and AT heavily inflated the type I error rates when participant compliance was affected by the participants’ general health.

**Conclusions:**

There are consequences for dichotomizing compliance information to make it fit into well-known methods. The results suggest the need for a method of estimating treatment effects that can utilize partial compliance information.

**Electronic supplementary material:**

The online version of this article (doi:10.1186/s13063-015-1044-z) contains supplementary material, which is available to authorized users.

## Background

Participant noncompliance is inevitable in clinical trials and must be considered by researchers in order to appropriately interpret data. Noncompliance exists when a participant does not comply with assigned treatment but remains in the study, diluting the differences between treatment groups and thereby affecting the power of a superiority study [1]. Thus, participant noncompliance is an issue that should be considered during both the design and analysis phases of a clinical trial. Methods have been developed to estimate a treatment effect in the presence of participant noncompliance.

The most common method employed to deal with noncompliance is intention to treat (ITT), which is considered by many to be the gold standard [2]. ITT simply analyzes participants as randomized, regardless of the actual treatment received. The drawback to ITT is that it may not measure the true effect of the treatment being studied [3]. Alternatives to ITT that attempt to find the true treatment effect include the per protocol (PP), as treated (AT), and instrumental variables (IV) methods. All three methods assume compliance is measured dichotomously but may be adapted for use when compliance is measured on a continuous scale. However, employing these methods in this manner may affect their estimation of the treatment effect.

Many increasingly complex methods of estimating treatment effects in the presence of noncompliance have been proposed. Imbens and Rubin proposed a method that imputes a participant’s latent compliance status (the participant’s possible compliance with either treatment) using Bayesian methods [4]. This work was extended to partial compliance by Jin and Rubin [5]. Nagelkerke et al. proposed adding an indicator of the treatment received and the residuals from a regression of the treatment received on the treatment assigned into regression models [6]. Efron and Feldman reframe the issue of partial compliance into a dose–response setting, but have the added complexity of participant self-selected doses as opposed to the randomly assigned doses employed in typical dose–response trials [7]. However, these methods are incredibly complex theoretically and in practice, and they may be too daunting for a researcher who has not extensively studied them to employ.

Other issues with compliance may arise depending on the design of the trial. One design that can lead to a unique compliance situation is the factorial design, in which participants are assigned to treatment levels for multiple therapies simultaneously. Since some participants are asked to receive multiple therapies, a factorial design can place a heavier burden on participants than a study of only one therapy. Furthermore, the experimental treatments being studied may place a heavier burden on participants than the control. In this case, a factorial design may compound the burden placed on the participants, especially among those randomized to multiple active treatment arms. Placing participants under higher burdens can have the expected effect of increasing noncompliance to treatment assignment for all the treatments in the study.

In this paper, we will assess the performance of the ITT, PP, AT, and IV methods for estimating a treatment effect in the presence of noncompliance measured on a continuous scale using simulations. Additionally, the performance of ITT, PP, AT, and IV will be assessed when employed in a two-by-two factorial design that places heavier burdens on participants in the experimental arms.

## Methods

### Noncompliance methods

Suppose that a clinical trial is designed to test the difference in two means with the hypotheses *H*_0_ : *μ*_0_ = *μ*_1_ versus *H*_*a*_ : *μ*_0_ ≠ *μ*_1_. If there is participant noncompliance in the study, then a noncompliance method must be employed in order to determine whether to reject the alternative hypothesis. ITT, PP, AT, and IV are four such methods that can be employed to test the hypothesis.

The ITT method is employed by simply disregarding whether a participant complied with assigned treatment and analyzing the participant as if he/she had fully complied with the assigned treatment. Using ITT, the estimation of the mean treatment effect is then the difference in the average outcomes (*δ*) of the participants who were assigned to the treatment and the participants assigned to the control:

1 $$ {\delta}_{ITT}={\mu}_1-{\mu}_0 $$

ITT is very simple to implement while preserving the randomization employed by the trial. Since ITT groups participants as they are randomized (and hence by assigned treatment), when there is noncompliance among the treated group, this estimate is not a true measure of the treatment effect. Instead, ITT measures the effect of treatment assignment on the outcome of interest [3].

The PP method removes noncompliant participants from the analysis, which ensures that all participants included were fully compliant to their assigned treatment. Thus, the PP estimate of treatment effect is as follows:

2 $$ {\delta}_{PP}={\mu}_{1c}-{\mu}_{0c} $$

where *μ*_1*c*_ is the average outcome for participants assigned to the experimental treatment who complied with the treatment. *μ*_0*c*_ is defined similarly for those in the control group.

AT deals with noncompliance by grouping participants for analysis based on the treatment actually received and ignores assigned treatment. Assuming that noncompliance only occurs when the participant receives the other treatment group’s therapy, the AT estimate of treatment effect is then determined as follows:

3 $$ {\delta}_{AT}=\frac{\mu_{1c}+{\mu}_{0n}}{2}-\frac{\mu_{0c}+{\mu}_{1n}}{2}, $$

where *μ*_1*n*_ is the average outcome of the participants who were assigned to the experimental treatment and did not comply. *μ*_0*n*_ is the average outcome of the noncompliant participants in the control arm of the study, whereas *μ*_1*c*_ and *μ*_0*c*_ are defined as above.

Both PP and AT attempt to estimate the true treatment effect using intuitive and easy to implement methods; however, both are problematic from an analytical perspective. Unless participant noncompliance is completely random, employing PP will remove a sub-population of the study population for whom the treatment may perform poorly (or perhaps better). Thus, the PP method may lead to biases in estimates, in addition to the possibility of introducing imbalances of covariates between treatment groups. AT explicitly breaks the randomization scheme of the study and allows the non-compliant participants to effectively self-select their treatment, which also introduces the possibility of biases in the estimation [8].

Another method for estimating the treatment effect in the presence of noncompliance is the instrumental variables (IV) method. Briefly, an instrumental variable is a variable that does not directly affect the outcome of interest except through the predictor of interest. In the application of the IV method, treatment assignment is considered an instrumental variable affecting the outcome of interest only through its impact on the treatment the participant actually receives. By using an instrumental variable, the approach attempts to remove the effects of the confounders that affect both a participant’s compliance and outcome [9]. In practice, this method inflates the ITT estimate by a factor of the estimated proportion of latent compliers (participants who would have complied with either treatment) in the study [8]:

4 $$ {\delta}_{IV}=\frac{\mu_1-{\mu}_0}{{\widehat{\pi}}_c}, $$

where ${\widehat{\pi}}_c$ is the estimate of the proportion of latent compliers in the study,

5 $$ {\widehat{\pi}}_c=1 - \frac{n_{1n}}{n_1}-\frac{n_{0n}}{n_0}, $$

*n*_1_ is the total number of participants in the experimental group and *n*_1*n*_ is the number of non-compliant participants in the experimental group. *n*_0_ and *n*_0*n*_ are defined similarly for the control group. Since the IV method employs the ITT estimate, participants remained grouped as randomized, so it avoids the problems inherent in the PP and AT methods. The IV method does have its own drawbacks though, such as that one of the assumptions for employing IV is that noncompliance only occurs in one direction [9]. That is, participant noncompliance will consist of either switching on to the experimental treatment for those in the control group or switching off of the experimental treatment from the experimental group, but not both.

PP, AT, and IV all assume that compliance to assigned treatment is binary: either a participant is or is not compliant. This may be a reasonable way to model compliance in studies of some therapies, but many treatments studied in clinical trials are administered multiple times over a period. Drug and behavioral interventions are common examples of such therapies. In these cases, it is possible for a participant to be neither fully compliant nor fully noncompliant to the treatment assignment, but merely partially compliant. Thus, in order for PP, AT, and IV to be employed as methods for estimating the treatment effect in the presence of partial compliance, participants’ compliance status must first be dichotomized. This is generally done by choosing a level of partial compliance (such as 80 %), above which, a participant is considered compliant, and below which, he is considered noncompliant. Dichotomizing the compliance level of participants may have a steep cost through a loss of information, which may lead to a loss of power and thus introduce bias into the estimate of treatment effect.

### Simulation

In order to assess the effect of dichotomizing compliance on the estimation of treatment effect when ITT, PP, AT, and IV are employed, we simulated two-arm studies (N = 128) in which some participants were partial compliers. We assumed normally distributed outcomes with a shared variance of *σ*^2^ = 2 and a true treatment effect of δ = 1. The sample size was chosen for a balanced study able to detect the treatment effect with at least 80 % power. We assumed participants assigned to the control arm of the study did not have access to the experimental treatment, and hence, the only noncompliance observed was in the experimental arm of the study. For each participant in the experimental arm of the study, the level of compliance for each participant was drawn from a distribution with possible values ranging from 0 to 1.

Four different compliance situations were examined using different distributions of the compliance: three Beta distributions (α = 1 β = 0.1765, α = 0.25 β = 0.05, and α = 0.5 β = 0.5), and a uniform distribution on [0,1]. An additional file shows the distribution of samples from each of the four distributions (see Additional file 1). The Beta (1, 0.1765) and Beta (0.25, 0.05) distributions represented situations where compliance was reasonably good (with expected compliance of 85 % and 83 %, respectively), as well as heavily clustered around 0 (complete noncompliance) and 1 (complete compliance). The Beta (0.5, 0.5) distribution and the uniform distribution both represented situations where the average proportion of noncompliance was much higher. In addition, because these two different distributions have the same expected compliance level (50 %) but different shapes, a comparison of these two compliance situations provided insight into the performance of the methods beyond how they respond to increased noncompliance. It was assumed that the effect of the experimental treatment was in proportion to the amount of treatment received as a function of compliance, so a participant assigned to the active treatment arm that only received 50 % of the assigned treatment (that is, 50 % compliant) had an expected change in outcome of 0.5.

To dichotomize the participants’ partial compliance, cutoff points were chosen above which the participant was considered compliant to the assignment. A wide range of cutoff points were considered for each set of simulated data: 0.5, 0.55, 0.6, 0.65, 0.7, 0.75, 0.8, 0.85, and 0.9.

After the participants were grouped as compliant or noncompliant based on the cutoff point, the four methods of interest were then applied to estimate the treatment effect from the simulated data. ITT, PP, and AT were simply applied as described above. The IV estimate was computed by dividing the ITT estimate by the proportion of participants labeled as compliant in the experimental group, which is an estimate of the proportion of latent compliers.

The average bias and mean squared error (MSE) were calculated for each of the methods, for each combination of cutoff point and compliance distribution. Furthermore, a two-tailed t-test was used to test the null hypothesis of no treatment difference. The power of the test was calculated as the proportion of the simulations for which the null hypothesis was rejected in favor of the alternative. The average bias, MSE, and power were assessed using 1000 replications. In order to assess the type I error rate, the simulations were repeated with no treatment effect (δ = 0). The type I error rate was then the proportion of simulations for which the null hypothesis was rejected. Type I error was assessed using 5000 replications.

Since in the above simulation, participant compliance was sampled completely independently of other factors, the assumptions required for PP and AT to be unbiased are met. Another set of simulations were run in order to assess the performance of all the methods when participant compliance is not independent. In this simulation, an untreated outcome for all participants was sampled from a normal distribution with a mean of 0 and variance 2. Participants with untreated outcomes below the 25^th^ percentile were presumed to be sicker, and their compliance was sampled from distribution with a lower mean than those with higher untreated outcomes. The healthy group’s mean compliance was 0.8, whereas the sicker group had a mean of 0.7. The variance of the compliance in both groups was 1/12. The results of this simulation were collected similarly to the previously described simulation.

The effect of the increased noncompliance resulting from increased burden in factorial designs was assessed by simulating a balanced 2 × 2 factorial design with a sample size of 128, chosen so that there was 80 % power to detect a treatment effect of specified size in one arm. Since we were interested in how the noncompliance in the more heavily burdened treatment group affected the estimate of treatment effect in comparison to a simple two-arm study, it was assumed that only one of the treatments being investigated in the factorial design had an effect on the participants’ outcome. Furthermore, it was assumed that the association between treatment level and outcome was not a function of the treatment level of the other treatment (that is, no treatment interaction). Participant outcomes were sampled from the normal distribution with a shared variance of 2 and a true treatment effect of 1, similar to the two-arm study simulation.

To represent increased noncompliance because of the increased burden placed on participants, the distribution of compliance depended on the treatment group. As in the two-arm simulation, it was assumed that participants assigned to the control group of the therapy of interest could not switch on to the experimental treatment and were thus fully compliant. Compliance for participants that were assigned to the experimental arm of the treatment of interest and the control arm of the other therapy was sampled from a Beta distribution with a mean of 0.9 and variance 1/12 representing relatively high compliance to assigned treatment. Participants in the experimental arms of both studies were placed under a heavier burden, and thus the compliance distributions were also Beta distributions with lower means to represent the potentially lower compliance. Three different situations were considered: small (mean = 0.8), larger (mean = 0.6), and extreme (mean = 0.4) drops in compliance, relative to those in a single treatment arm (mean = 0.9). The variance of the increased burden compliance distributions were all 1/12.

The partial compliance was dichotomized in this situation using the same range of cutoff points as in the two-arm simulation. ITT, PP, AT, and IV were then employed as described previously to estimate the treatment effect of the therapy of interest. Average bias and MSE of the estimates were calculated, and the power and type I error rate of the test of significance of the estimate of treatment effect were assessed as they were in the two-arm simulations. A total of 1000 replicated datasets for each situation were generated in order to calculate the average bias, MSE and power. A total of 5000 datasets were generated to calculate the type I error rate.

## Results

### Two-arm study, compliance independent of disease burden

**Fig. 1:**
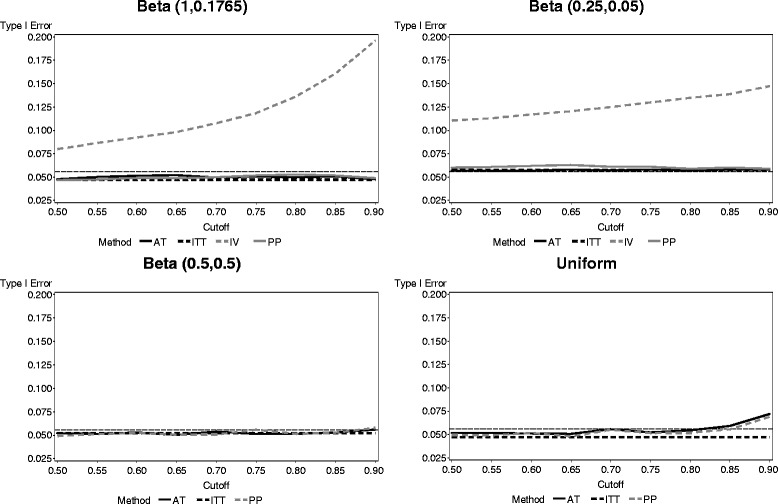
Type I error rate of each noncompliance method across four compliance distributions (reference line provides result for fully compliant population)

The type I error rate for each test when δ = 0 is presented in Fig. 1. The ITT method controlled the type I error rate very well, staying very close to the nominal level of 0.05. In all cases, except those for which the cutoff point was close to 1, the PP and AT methods also controlled the type I error rate reasonably well. On the other hand, the IV method inflated the type I error rate; in some cases, the inflation was extreme. For the compliance distributions with a mean of 0.5, the results of the IV method are not pictured in order to keep the figure to an appropriate scale. For both distributions with mean 0.5 and all cutoff points, the type I error rate with the IV method never fell below 30 % and rose as high as nearly 85 % in the case of uniform compliance with a cutoff of 0.9.

**Fig. 2:**
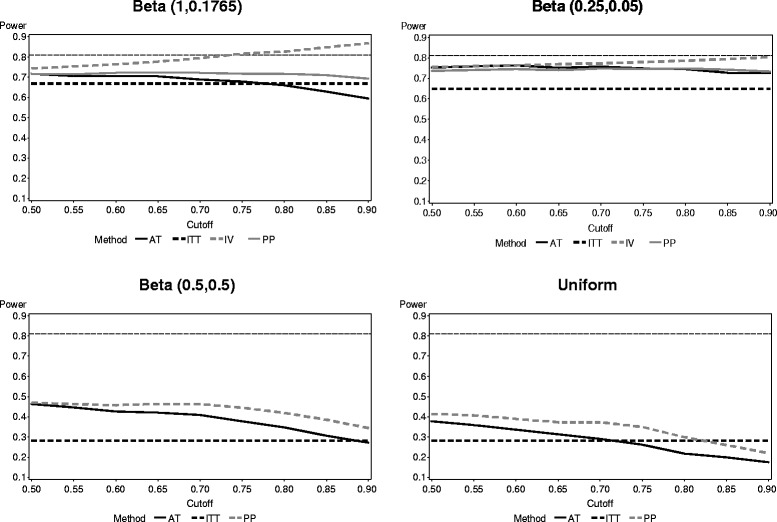
Power of each noncompliance method across four compliance distributions (reference line provides result for fully compliant population)

The power of the test for each method is presented in Fig. 2. The power for all four methods was tied to the amount of noncompliance in the study. When employing ITT, the power was significantly reduced when a large amount of noncompliance (for example, 50 % noncompliance) was present in the study. Even when compliance was better in the study though, ITT still had lower power than the other three methods. The IV method generally had the highest power of the methods, and it increased as the cutoff point approached 1. PP generally had higher power than AT, but both performed relatively similarly in comparison to ITT and IV; both PP and AT had only slightly higher power than ITT.

**Fig. 3:**
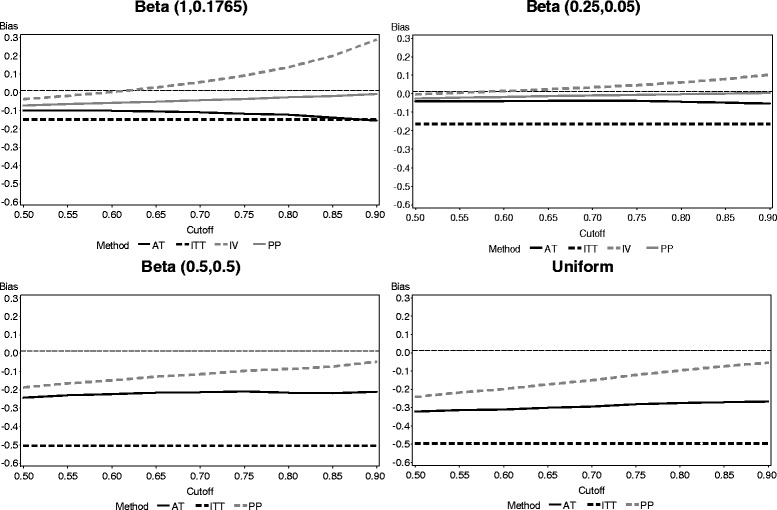
Biases of each noncompliance method across four compliance distributions (reference line provides result for fully compliant population)

The average bias of each of the methods for the different compliance distributions in the two-arm study simulation is presented in Fig. 3. The same general relationship between the four methods was preserved across all compliance distributions. As expected, ITT consistently underestimated the true treatment effect and was unaffected by the choice of cutoff point for dichotomization (since it did not take compliance into account). The bias of the ITT estimate was affected by the mean level of compliance in the study population, but not the shape of the distribution. Hence, the bias of ITT was similar for both the uniform distribution and the Beta (0.5, 0.5) distribution. Both PP and AT also consistently underestimated the treatment effect as well, but both were closer than ITT. The average bias of both PP and AT was also relatively static with regard to the cutoff point chosen by the investigators. The IV estimator performed quite differently. IV generally overestimated the treatment effect, and the bias increased exponentially as the cutoff point approaches 1. The strength of the IV estimate’s bias varied greatly depending on the distribution of the compliance. IV performed better when the distribution of the compliance is clustered around 0 and 1 (that is, there was generally a clear distinction between a “complier” and a “noncomplier”).

The MSE of the methods in each compliance distribution in the two-arm study simulation is presented in Table 1. The IV estimate consistently had the highest MSE of the four methods, while also increasing exponentially as the cutoff point approached 1. The ITT method generally had the second highest MSE, while AT and PP performed similarly to each other. It can be noted that the MSE for both AT and PP do increase as the cutoff point approaches 1, but not nearly at the rate at which IV increased.

**Table 1:** Relative mean squared error (MSE) by method, cutpoint, and compliance distribution (relative to intention to treat (ITT))

Cutpoint	Method	Compliance distribution			
		Beta (1, 0.1765)	Beta (0.25, 0.05)	Beta (0.5, 0.5)	Uniform
0.9	PP	1.064	0.951	0.991	2.251
	AT	1.148	0.898	1.023	2.365
	IV	2.598	1.508	17.183	173.917
0.85	PP	1.024	0.930	0.863	1.390
	AT	1.081	0.868	0.905	1.504
	IV	2.004	1.434	8.887	44.182
0.8	PP	0.998	0.915	0.760	1.050
	AT	1.025	0.847	0.793	1.160
	IV	1.671	1.366	5.543	18.681
0.75	PP	0.985	0.918	0.690	0.866
	AT	1.001	0.856	0.712	0.981
	IV	1.469	1.313	3.833	9.308
0.7	PP	0.954	0.913	0.658	0.795
	AT	0.959	0.853	0.682	0.912
	IV	1.336	1.274	2.858	5.467
0.65	PP	0.944	0.909	0.637	0.720
	AT	0.944	0.851	0.656	0.835
	IV	1.242	1.245	2.230	3.476
0.6	PP	0.933	0.900	0.616	0.667
	AT	0.927	0.845	0.635	0.779
	IV	1.172	1.218	1.818	2.369
0.55	PP	0.933	0.898	0.607	0.651
	AT	0.927	0.848	0.619	0.745
	IV	1.127	1.190	1.555	1.763
0.5	PP	0.931	0.904	0.592	0.641
	AT	0.924	0.857	0.603	0.720
	IV	1.092	1.171	1.362	1.386

Comparing the uniform compliance situation with the Beta (0.5, 0.5) situation, the PP, AT, and IV methods all performed better in the Beta (0.5, 0.5) situation where participant compliance tended to either 0 or 1. The results for ITT are similar to each other for both compliance distributions since they have the same mean level noncompliance. The bias in the PP and AT methods was similar in each situation, but each method experienced higher variance (reflected in the increased MSE) in the uniform distribution. The higher variance predictably led to lower power and higher type I error rates for both methods, especially at higher cutoff points. The IV method had similar results as PP and AT in comparing the two compliance situations, but the increase in variance of the estimate was much more dramatic. Additionally, the IV method did have a notable increase in bias in the uniform compliance situation when compared to the Beta (0.5, 0.5) situation.

### Two-arm study, compliance as a function of disease burden

**Fig. 4:**
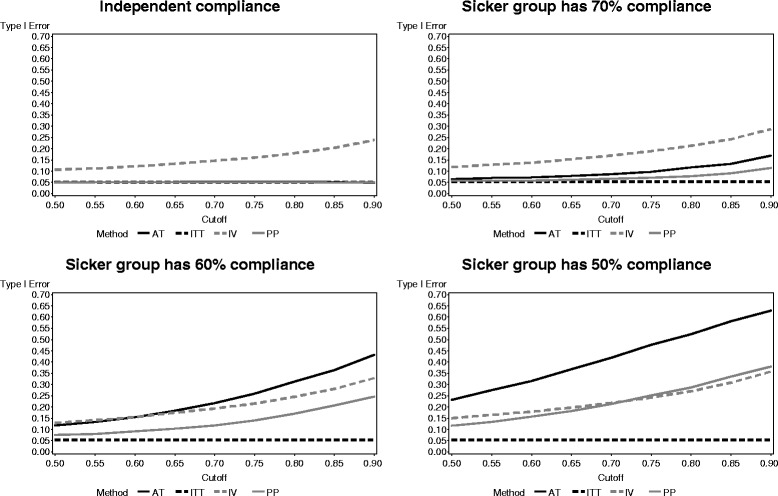
Type I error rates of each noncompliance method when compliance is related to untreated outcomes

The type I error rates when the sicker participants had lower compliance levels are presented in Fig. 4. ITT continues to maintain a type I error rate close to 0.05, while the IV method has inflated type I error rates as before. However, PP and AT both have inflated type I error rates that grow more extreme as the compliance level for the sicker group drops and the cutoff point increases, with AT having higher type I error rates than PP. Both methods even have type I error rates greater than that of IV as compliance among the sicker group drops.

**Fig. 5:**
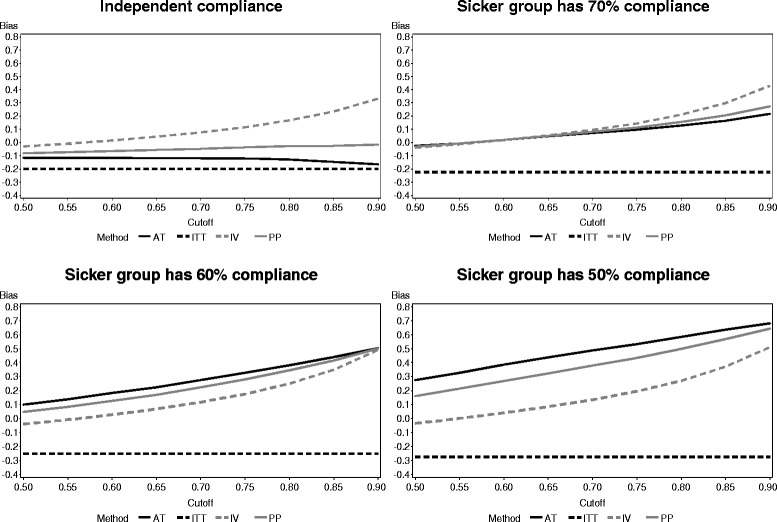
Biases of each noncompliance method when compliance is related to untreated outcomes

Figure 5 presents the biases of each method when sicker participants experience lower compliance. Once again, ITT and IV perform similarly to the first simulation, underestimating and overestimating the treatment effect respectively. In this situation though, PP and AT generally overestimate the treatment effect as opposed to underestimating it in the first simulation. Both overestimate the bias to a greater degree in the situations with higher cutoff points and lower compliance among the sicker participants.

### Factorial design

**Fig. 6:**
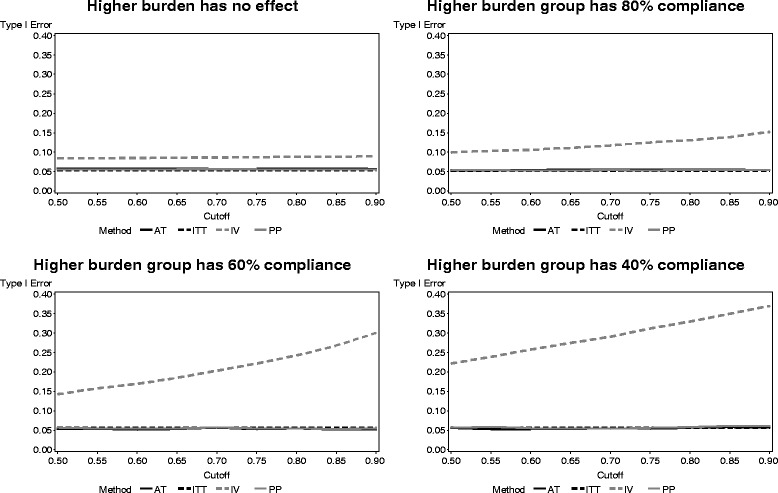
Type I error of each noncompliance method across participant burden levels

The results of the type I error rate for each method with higher burden due to being assigned the experimental treatment in both arms of a factorial study are presented in Fig. 6. The ITT, PP, and AT are similar regardless of the level of compliance for the participants experiencing higher burden from multiple active treatments. The IV method had drastically increased type I error rates as the compliance in the increased burden group decreased.

**Fig. 7:**
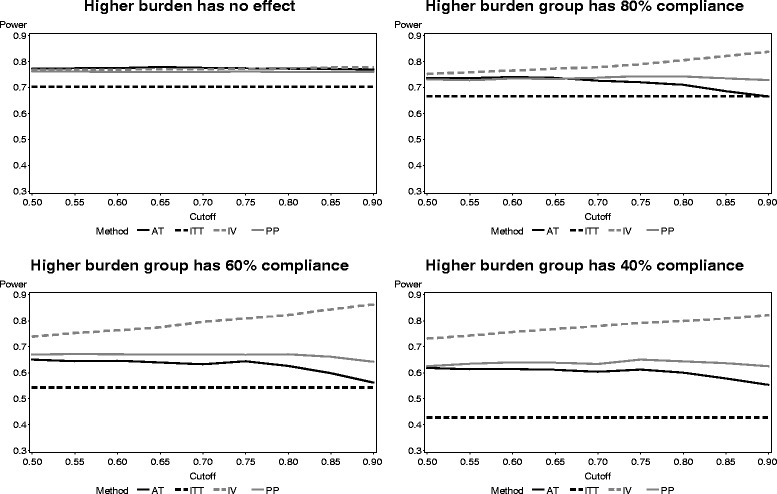
Power of each noncompliance method across participant burden levels

The power of each method in the factorial study simulation is presented in Fig. 7. The effect of higher participant burden on ITT was very strong, with power dropping to about 40 % when the burden was extremely heavy. The PP and AT methods also have reduced power as the compliance of the participants in the higher burden group decreases, but both have higher power than ITT in each situation observed. The IV method is less affected by the level of compliance in the higher burden group than the other methods. As compliance drops, the power when using the IV method may be reduced either when a low cutoff point is used or increased when a high cutoff point is used.

**Fig. 8:**
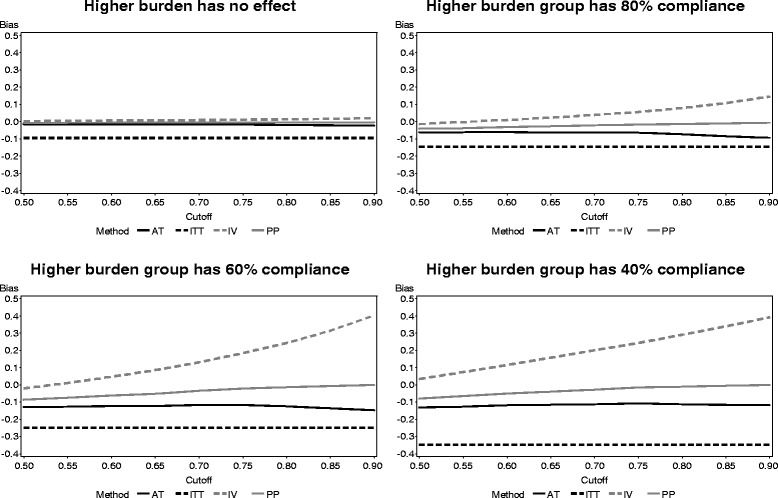
Bias of each noncompliance method across participant burden levels

The effect of higher participant burden in the factorial study simulation on the bias of each method is presented in Fig. 8. The bias of the ITT method is affected by the higher burden, decreasing as participants on two active treatments experience lower levels of compliance. The IV method appears to experience increased positive bias as compliance in the higher burden group decreases. However, the biases of both PP and AT are comparatively not affected by the amount of compliance in the increased burden group.

Table 2 presents the relative MSE results for the factorial study simulation. The PP and AT methods generally had smaller MSE than ITT. Higher burden levels further widened the gap between the ITT method and the AT and PP methods. In comparison, the MSE using the IV method is higher than the ITT method’s MSE with the difference accentuated by increasing participant burden.

**Table 2:** Relative mean square error (MSE) by method, cutpoint, and level of burden (relative to intention to treat (ITT))

Cutpoint	Method	Level of burden			
		Heavy	Medium	Mild	None
0.9	PP	0.788	0.982	1.052	1.028
	AT	0.749	0.983	1.052	1.017
	IV	2.977	3.269	1.710	1.184
0.85	PP	0.776	0.950	1.019	1.028
	AT	0.742	0.933	1.014	1.009
	IV	2.647	2.629	1.531	1.175
0.8	PP	0.752	0.909	1.006	1.020
	AT	0.711	0.886	0.994	1.009
	IV	2.362	2.195	1.421	1.175
0.75	PP	0.729	0.905	1.000	1.028
	AT	0.690	0.886	0.979	1.015
	IV	2.110	1.894	1.347	1.167
0.7	PP	0.715	0.883	1.001	1.021
	AT	0.679	0.865	0.984	1.015
	IV	1.915	1.647	1.289	1.159
0.65	PP	0.694	0.870	0.996	1.021
	AT	0.662	0.858	0.985	1.008
	IV	1.728	1.468	1.243	1.152
0.6	PP	0.686	0.860	0.985	1.013
	AT	0.656	0.852	0.970	1.008
	IV	1.560	1.336	1.213	1.144
0.55	PP	0.669	0.859	0.981	1.014
	AT	0.636	0.856	0.971	1.001
	IV	1.413	1.235	1.179	1.144
0.5	PP	0.665	0.854	0.970	1.013
	AT	0.629	0.842	0.957	1.000
	IV	1.291	1.158	1.147	1.144

## Discussion

The results of the simulation study in the two-arm case reveal why ITT has endured as the assumed gold standard method of estimating a treatment effect in the presence of participant noncompliance in a superiority trial. The ITT method is always biased towards the hypothesis of no treatment effect, which is commonly the null hypothesis of the study question. In this case, employing ITT to estimate the treatment effect will not inflate the type I error rate of the test. This great benefit of ITT comes at the cost of the power of the study, however. The strength of the bias of ITT, and hence the amount of the loss of power in the study, is directly tied to the amount of noncompliance in the study. If a study has only a small amount of noncompliance, the loss of power may be negligible; but if enough noncompliance is present, the loss of power may be drastic. PP and AT provided a little benefit over employing ITT in any of the situations observed. In fact, both of the methods may perform poorly in comparison to ITT in some situations when the cutoff point was close to 1, and both have the ability to introduce additional biases into the analyses.

The clearest conclusion from the results of the simulations is that employing the IV method with partial compliance by first dichotomizing the compliance measure is inappropriate. In this situation, IV will consistently overestimate the treatment effect for most cutoff points that are greater than 0.5. Additionally, in a truly dichotomous compliance setting, IV generally has the highest variance of these four methods [4], and that remains true in the partial compliance setting as well. These issues with IV mean that testing with this method will lead to high power, but also high type I error rates, and thus it cannot be recommended that IV be used in this manner.

The decision of where the cutoff point is for dichotomizing a collected partial compliance measure in order to employ PP, AT, or IV can have a strong effect on the estimation. It is clear that if “compliance” is defined stringently by choosing the cutoff point to be close to 1, all three methods will perform poorly. In addition, the distribution of participant compliance plays an important role in the performance of the three methods that dichotomize the compliance. PP, AT, and IV all had better performance under compliance distributions that are clustered around 0 and 1. Since dichotomizing under these compliance distributions is more natural, this result is not unexpected. The effect of clustering in the distribution on the methods’ performance is best seen by comparing the uniform distribution compliance with the Beta (0.5, 0.5) distribution compliance. Both distributions have the same mean compliance level of 0.5, but the Beta distribution has clustering around 0 and 1. As we would expect, all the methods perform poorly under both compliance distributions because of the fairly high level of noncompliance, but PP, AT, and IV all perform worse under the uniform distribution than the Beta distribution.

While the performance of both PP and AT is reasonable when compliance is independent of other factors, the situation when compliance is related to other patient characteristics is much worse. In the simulated situation, PP and AT will tend to remove or regroup participants in the experimental arm with lower outcomes since they will also generally have lower compliance as well. This inflates the average outcome in the experimental arm and leads to overestimation of the treatment effect and that in turn to inflated type I error rates. Since participant compliance being completely independent to other participant characteristics is highly unlikely, the use of PP and AT will likely provide suspect results.

The effect of increased participant burden is straightforward. The issues that each method experienced in the two-arm study are intensified. Hence the ITT method’s power drops precipitously, and the IV method’s type I error inflation becomes more drastic as the participant burden increases. PP and AT perform relatively well in this setting, but there is still a loss of power incurred when the burden increases even for these methods.

These simulations assumed that only participants in the experimental arm could truly be noncompliant. This means that control arm participants did not have access to the experimental treatment and that compliance to any placebo being used in the control arm had a negligible effect on participants’ outcomes. However, if the experimental treatment is freely available, control arm noncompliance by switching onto the experimental treatment may occur. This study did not investigate these situations, but it is assumed that similar results that are heightened by the average treatment effect of both treatment arms being affected by participant noncompliance may occur.

Simulations in this study assumed that there is a linear relationship between the level of compliance to the active treatment and the observed outcome, but possibly, for some treatments, the relationship may be different. Different relationships may alter the effect of the choice of cutpoint for dichotomization on the results. For example, if the effect of the treatment plateaus at 80 % compliance, then 80 % would be a natural cutpoint for dichotomization. In this case, more stringent definitions of compliance may have even stronger negative consequences than what is observed in the simulations presented in this paper. Further research into the different relations between compliance and observed outcomes would provide greater understanding into the role the relation plays in the performance of methods for dealing with noncompliance.

Dichotomization is the most extreme method of categorizing continuous compliance data. Categorizing into more than two groups may reduce the strength of the effect of choosing cutpoints in the results, as well as being able to more accurately represent a wider variety of compliance distributions. However, categorizing compliance into more than two groups is very rarely discussed in the literature. The development of statistical methods that can use such categorization of compliance are a possible avenue for future research.

## Conclusions

Measures of participant compliance to assigned treatment that are collected in a clinical trial are often continuous values. However, the most commonly known methods for addressing noncompliance in a trial assumed that compliance is binary. This paper used simulations to investigate the performance of ITT, PP, AT, and IV after dichotomizing partial compliance measures. The results of the simulations indicated that the use of PP and AT provides little benefit to estimating treatment effect over the gold standard of ITT when used with dichotomized compliance data, while using the IV method with dichotomized data often led to unacceptably inflated type I error rate. Choosing a stringent definition of compliance may also lead to inflated type I error rates for the PP and AT methods, especially if the distribution of the compliance does not cluster around 0 or 1. Simulations investigating the possible of effects of reduced compliance for participants on two active treatments in a factorial design study were also created. The results of the simulations are similar to the results in a two-arm trial. The increased burden for participants in a factorial design study mainly affected the results through the increased level of overall noncompliance in the study population.

No statistical method can improve on keeping participants compliant, and researchers must take care during the design and execution of their trial to address compliance issues before or as they arise. When noncompliance occurs though, it is important to choose a method that is appropriate for the situation at hand. The results of this study suggest that methods that can account for the partial compliance information should be an improvement over adapting the information to fit into well-known methods not designed for the situation. These issues apply to employing noncompliance methods in the factorial design setting as well. The investigation and development of methods designed for use in both partial compliance and factorial design settings is the subject of future work.

## Additional file

Additional file 1:
**Distributions of the Four Compliance Distributions.** Bar graphs of samples from each of the four compliance distributions used in the two-arm trial simulation. (DOCX 82 kb)

## Abbreviations

AT: as treated
ITT: intention to treat
IV: instrumental variable
MSE: mean squared error
PP: per protocol
