# Supplementary figures and images for: Dichotomizing partial compliance and increased participant burden in factorial designs: the performance of four noncompliance methods
Source: Trials. 2015 Nov 17;16:523. doi: 10.1186/s13063-015-1044-z (PMC4647702; doi:10.1186/s13063-015-1044-z)

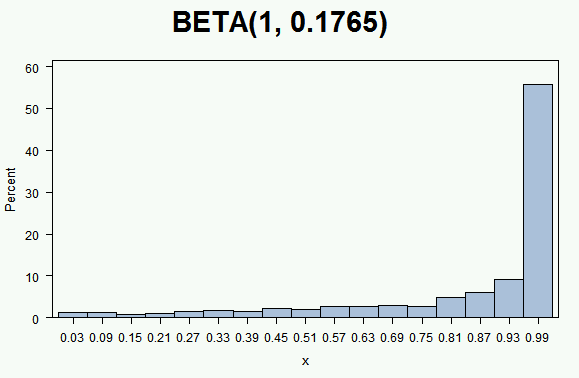


Mean=0.85 Variance=0.0595


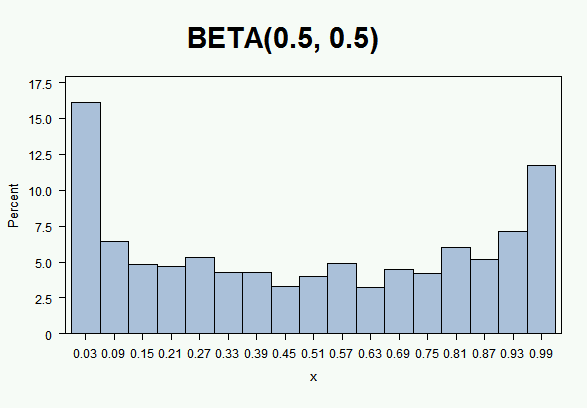


Mean=0.50 Variance=0.125


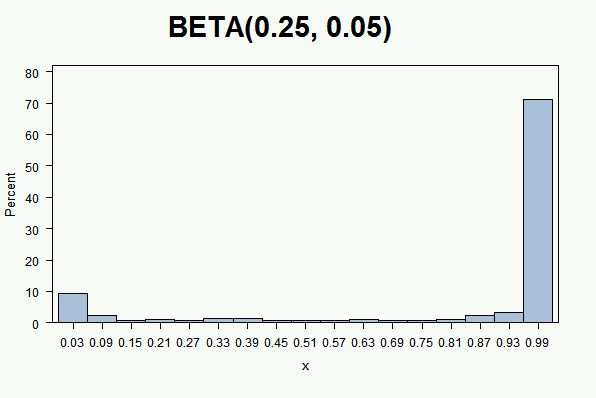


Mean=0.83 Variance=0.109


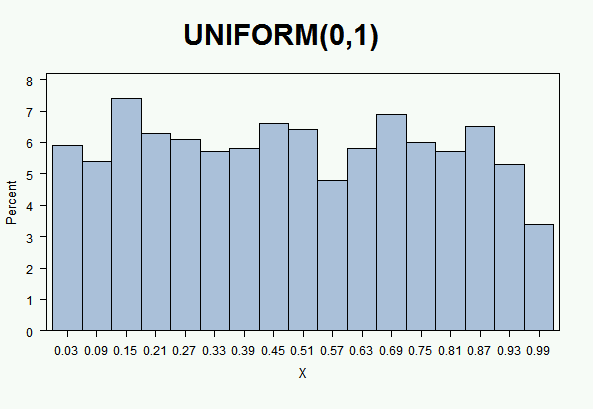


Mean=0.50

Variance=0.083

Supplement: Additional file 1: — Distributions of the Four Compliance Distributions. Bar graphs of samples from each of the four compliance distributions used in the two-arm trial simulation. (DOCX 82 kb) [file 13063_2015_1044_MOESM1_ESM.docx]
